# Response of Bacterial Metabolic Activity to Riverine Dissolved Organic Carbon and Exogenous Viruses in Estuarine and Coastal Waters: Implications for CO_2_ Emission

**DOI:** 10.1371/journal.pone.0102490

**Published:** 2014-07-18

**Authors:** Jie Xu, Mingming Sun, Zhen Shi, Paul J. Harrison, Hongbin Liu

**Affiliations:** 1 State Key Laboratory of Tropical Oceanography, South China Sea Institute of Oceanology, Chinese Academy of Sciences, Guangzhou, China; 2 Division of Life Sciences, The Hong Kong University of Science and Technology, Clear Water Bay, Kowloon, Hong Kong; Mount Allison University, Canada

## Abstract

A cross-transplant experiment between estuarine water and seawater was conducted to examine the response of bacterial metabolic activity to riverine dissolved organic carbon (DOC) input under virus-rich and virus-free conditions, as well as to exogenous viruses. Riverine DOC input increased bacterial production significantly, but not bacterial respiration (BR) because of its high lability. The bioavailable riverine DOC influenced bulk bacterial respiration in two contrasting ways; it enhanced the bulk BR by stimulating bacterial growth, but simultaneously reduced the cell-specific BR due to its high lability. As a result, there was little stimulation of the bulk BR by riverine DOC. This might be partly responsible for lower CO_2_ degassing fluxes in estuaries receiving high sewage-DOC that is highly labile. Viruses restricted microbial decomposition of riverine DOC dramatically by repressing the growth of metabolically active bacteria. Bacterial carbon demand in the presence of viruses only accounted for 7–12% of that in the absence of viruses. Consequently, a large fraction of riverine DOC was likely transported offshore to the shelf. In addition, marine bacteria and estuarine bacteria responded distinctly to exogenous viruses. Marine viruses were able to infect estuarine bacteria, but not as efficiently as estuarine viruses, while estuarine viruses infected marine bacteria as efficiently as marine viruses. We speculate that the rapid changes in the viral community due to freshwater input destroyed the existing bacteria-virus relationship, which would change the bacterial community composition and affect the bacterial metabolic activity and carbon cycling in this estuary.

## Introduction

Estuaries are in general a significant source of CO_2_ to the atmosphere, where CO_2_ efflux is estimated to be 0.25±0.25 Pg C y^−1^ at the global scale [1]. Low-latitude estuarine/coastal waters receive approximately two-thirds of the terrestrial organic carbon (OC) and have higher microbial decomposition rates due to higher temperatures [2]–[5]. Hence, it is speculated that more CO_2_ is degassing in lower latitude estuarine and coastal waters [1].

The Pearl River is subtropical and the second largest river in China, with an annual average freshwater discharge of 10,524 m^3^ s^−1^, which was calculated by *Yin et al.* [6] from *Zhao* [7]. About 80% of the discharge occurs in the wet season (April–September). High dissolved organic carbon (DOC) concentrations up to 473 µmol C L^−1^ occur in the Pearl River estuary, most of which originates from sewage [8]. However, the CO_2_ degassing flux is reported to be 6.92 mol C m^2^ y^−1^ in the entire Pearl River estuary [9], much lower than that (36.5–182.5 mol C m^2^ y^−1^) for the European estuaries [4], [10]. Similarly, low CO_2_ flux (only 8.1 mol C m^2^ y^−1^) has also been documented in the Hoogly estuary (NE India) that receives a huge amount (∼400 million tons yearly) of sewage [11]. One explanation for the surprisingly lower CO_2_ flux in these low-latitude estuaries is due to the large freshwater discharge and short residence time of the discharged water [12], [13]. To date, the underlying mechanism for the lower CO_2_ flux remains unknown.

The riverine DOC is primarily mineralized by heterotrophic bacteria that produce a significant amount of new biomass from DOC and transform DOC into inorganic carbon [14]–[16]. High terrestrial organic carbon input exerts a great influence on bacterial metabolism and community composition [17], [18]. Prior studies suggest that the lability of DOC plays a key role in regulating bacterial respiration rates [19]. Cell-specific bacterial respiration increases sharply in response to the decreasing quality of DOC since a larger percentage of energy is used for maintenance processes to safeguard metabolic flexibility at the cost of energetic efficiency [19], [20]. A recent study indicates that bacteria grown on sewage-derived DOC have high production and low respiration rates, while those on marine-derived DOC at have high respiration rates [21]. Hence, the lability of DOC could potentially affect CO_2_ degassing fluxes in high DOC estuaries.

In recent years, it has been recognized that viruses play an important role in regulating bacterial metabolic activities [22]–[24]. Bacterial production is reported to be reduced by up to 50% due to viral lysis [25]. However, little attention has been paid to interactions between bacteria and indigenous viruses and the effect of viruses on microbial decomposition of riverine DOC in estuaries, which may be important in understanding the fate of terrestrial organic carbon in the ocean. To our knowledge, only few studies have been conducted which suggested that the bacterial response to different sources of viruses may vary [26]–[28]. Therefore, a study on response of bacterial metabolic processes to riverine DOC and exogenous viruses is needed to determine the reasons for the lower CO_2_ degassing fluxes in the Pearl River estuary. A cross-transplant experiment of estuarine water and seawater was conducted to examine the response of bacterial metabolic activity to riverine DOC input under virus-rich and virus-free conditions. This study will increase our understanding of the role of bacteria in influencing riverine organic carbon cycling in estuarine waters.

## Materials and Methods

**Figure 1:**
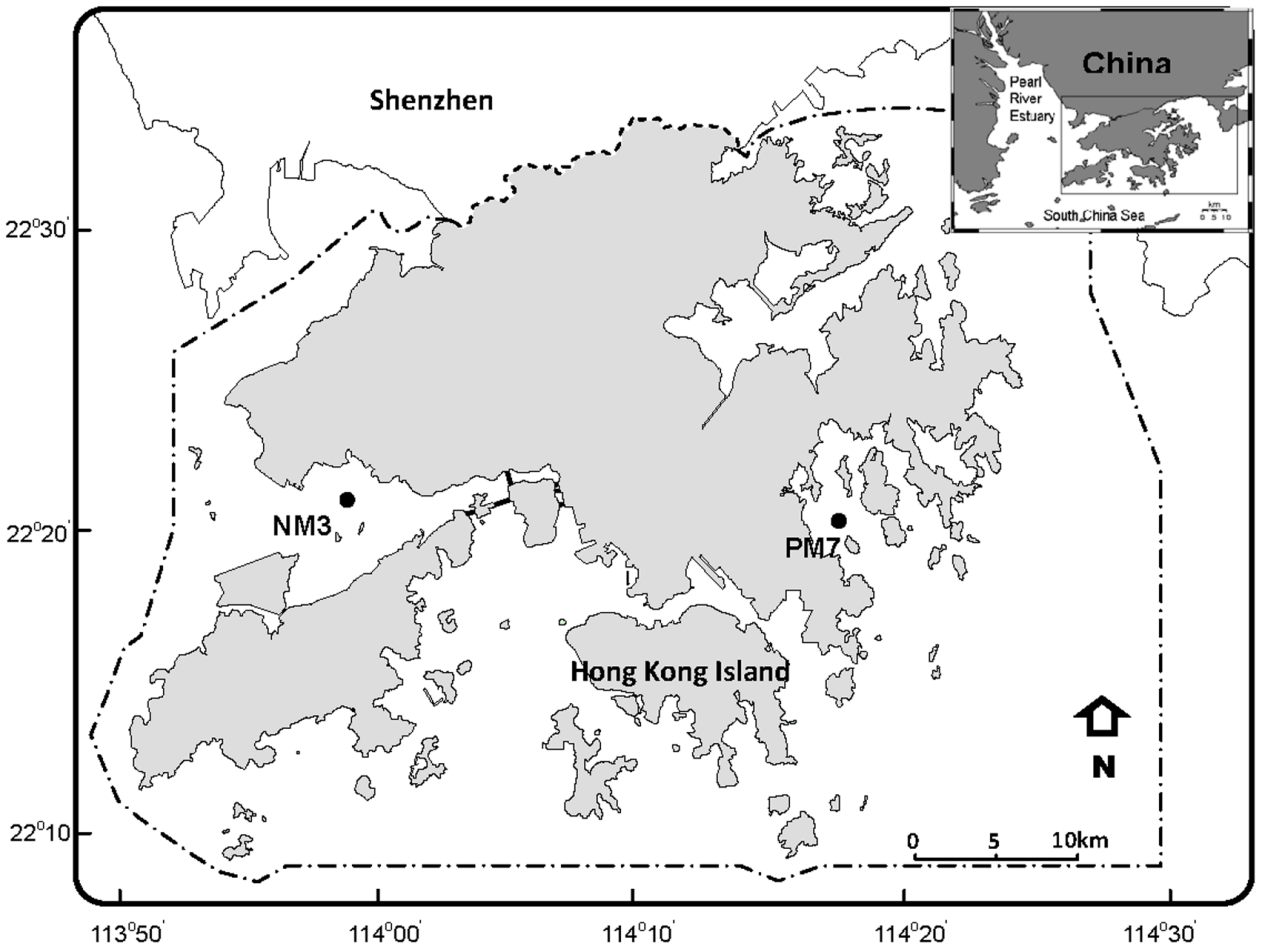
Location of the sampling stations in Hong Kong waters. These 2 stations are the same as the Environmental Protection Department of Hong Kong monitoring stations. The water depths for NM3 and PM7 are 15 and 17

No specific permissions were required for sampling at stations PM7 and NM3 in Hong Kong waters. We confirmed that the field studies did not involve endangered or protected species at these two stations. Seawater samples were taken from 1 m depth in August (wet summer season) 2013 at two contrasting coastal sites, a relatively pristine coastal station (PM7) with no influence from sewage or freshwater discharge, and a eutrophic estuarine station near the Pearl River (NM3) (Fig. 1). The water depths of PM7 and NM3 were 17 and 15 m, respectively. Samples for nutrients, dissolved organic carbon (DOC), and bacterial and viral abundance were taken.

### Experimental set-up

**Figure 2:**
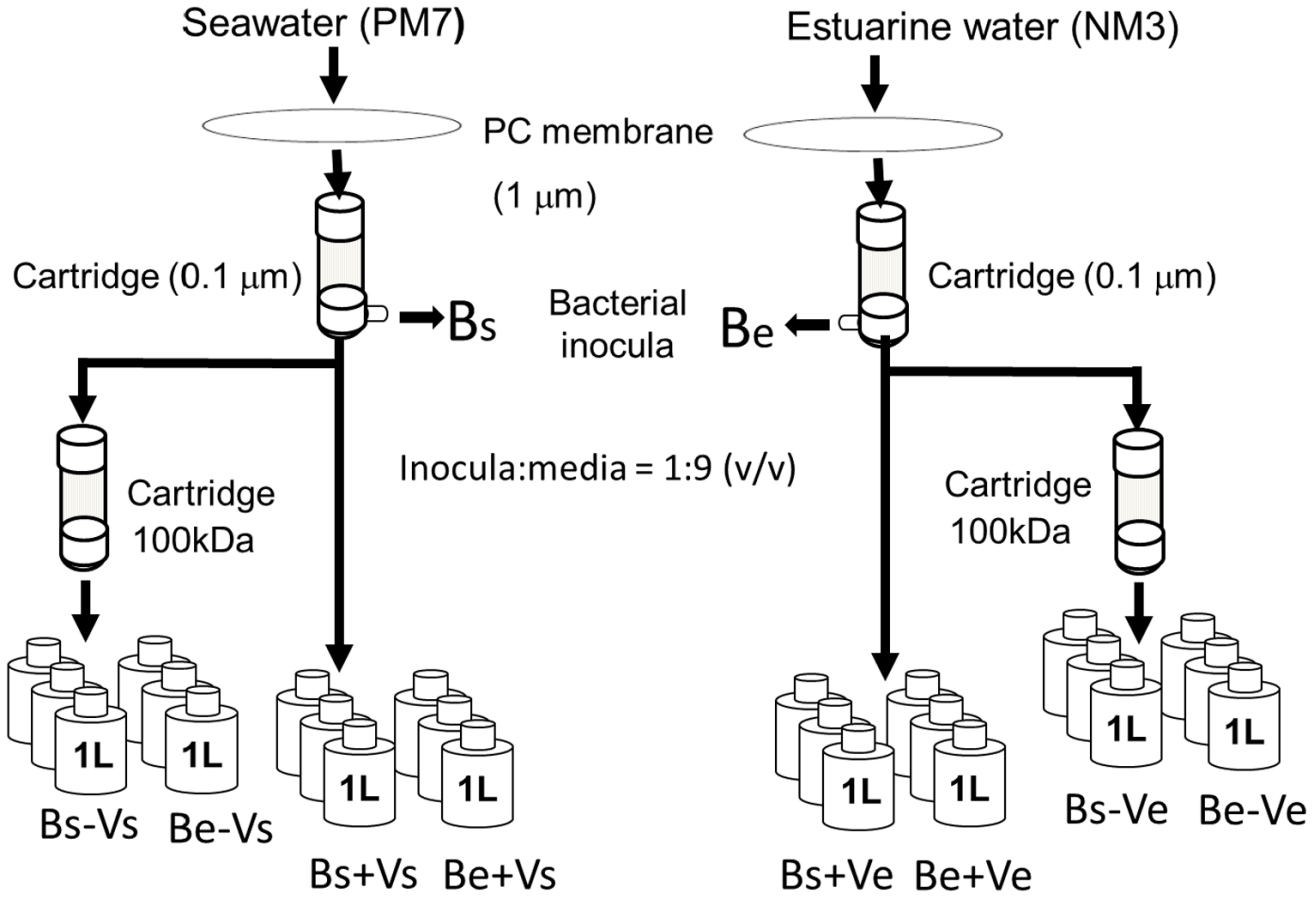
A schematic of the experimental setup. Bs and Be denote marine and estuarine bacteria, respectively. Vs and Ve denote marine and estuarine viruses, respectively.

Water samples were passed through a 1 µm polycarbonate membrane and the filtrate was used as the bacterial inoculum. Virus-rich water was obtained by filtration of water samples through a 0.1 µm cartridge. Relatively virus-free water was prepared by filtration of the 0.1 µm filtrate through a 100 kDa cutoff polysulfone cartridge (Millipore) using tangential flow ultra-filtration. About 600 ml of the bacterial inoculum was mixed with 5.4 L of virus-rich and relatively virus-free water from each station in acid-washed and Milli-Q rinsed polycarbonate carboys and distributed to triplicate glass bottles (1 L) to obtain eight treatments (Fig. 2) as follows: 1) estuarine bacteria + virus-rich estuarine water (Be+Ve); 2) estuarine bacteria + virus-free estuarine water (Be-Ve); 3) estuarine bacteria + virus-rich seawater (Be+Vs); 4) estuarine bacteria + virus-free seawater (Be-Vs); 5) marine bacteria + virus-rich estuarine water (Bs+Ve); 6) marine bacteria + virus-free estuarine water (Bs-Ve); 7) marine bacteria + virus-rich seawater (Bs+Vs) and; 8) marine bacteria + virus-free seawater (Bs-Vs).

Samples were incubated in the dark for 24 h. Running seawater was used to maintain the surface *in situ* temperature. Samples for nutrients, bacterial abundance (BA), bacterial production (BP), bacterial respiration (BR), viral abundance (VA) were taken at the beginning and end of the incubations. DOC samples were taken at the beginning of the incubation.

### Nutrients and DOC concentrations

Nutrient concentrations (NO_3_,
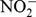
_,_ NH_4_, PO_4_ and SiO_4_) were determined colorimetrically with a SKALAR autoanalyser following the protocols described by *Strickland and Parsons* [29] and *Grasshoff et al.* [30]. Dissolved inorganic nitrogen (DIN) was the sum of NO_3_, 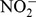
and NH_4_. Samples for DOC were filtered through a 0.2 µm acetate cellulose membrane and analyzed with a high temperature combustion method using a Shimadzu TOC-5000 analyzer [31].

### Bacterial and viral abundance, bacterial production, respiration and cell size

Samples for bacterial and viral abundance were taken in micro-centrifuge tubes, fixed with buffered paraformaldehyde (final concentration 0.5%), and then stored at −80°C until analysis by a flow cytometer (Becton-Dickinson FACSCalibur). Bacterial and viral abundance was determined according to the methods described by *Marie et al.* [32], [33] and *Xu et al*. [24], respectively. Bacteria were determined on a plot of green fluorescence vs. side scatter (SSC), which also provided information on the relative DNA content and cell size by the means of SYB-green fluorescence side scatter, respectively. The values for relative DNA content and cell size were calibrated with 1 µm sized beads.

The measurement of bacterial production (BP) and bacterial respiration (BR) was described previously [24]. BP was measured using ^3^H-leucine following the JGOFS protocol [31] and the final concentration of ^3^H-leucine was 35 nM. BP was calculated with the empirical conversion factor of 3 kg C mol leucine^−1^ [34]. Dissolved oxygen for bacterial respiration was titrated using an automated titration apparatus (716 DMS Titrino Metrohm) [35]. BR was presented in carbon units assuming that the respiratory quotient was 1 [36].

Bulk BP was calculated using the following equation:
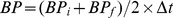
where BP_i_ and BP_f_ are bacterial production (µg C L^−1^ h^−1^) measured at the beginning and the end of the experiment, Δt is the time interval between the beginning and the end of the experiment.

The cell-specific BP (sBP) and BR (sBR) were calculated according to the following equations, respectively:
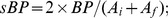


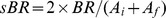
where A_i_ and A_f_ are the initial and final abundance of bacteria, respectively.

The growth rates of bacteria and viruses were calculated using the following equation:
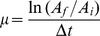
where A_i_ and A_f_ are the initial and final abundance of bacteria or viruses, respectively. Δt is the time interval between the beginning and the end of the experiment.

Bacterial growth efficiency was calculated using the following equation:
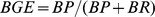



### Statistical analyses

Statistical analyses were performed using SPSS software. A parametric ANOVA analysis with an LSD (least square difference) multiple comparison technique was conducted to determine any significant difference between treatments (*p*<0.05). The error bars represent the standard error from triplicates for each treatment.

## Results

Surface salinity (22.40) at estuarine station (NM3) was much lower than that (28.64) at coastal station (PM7) (Table 1). Nutrient and DOC concentrations were high at NM3 and low at PM7. However, bacterial and viral abundance (2.45×10^9^ cells L^−1^ and 2.57×10^10^ particles L^−1^, respectively) at PM7 was higher than those (4.28×10^8^ cells L^−1^ and 9.40×10^9^ particles L^−1^, respectively) at NM3 (Table 1).

**Table 1:** Initial nutrient (DIN, PO_4_ and Si(OH)_4_) and DOC concentrations, bacterial abundance (BA), and viral abundance (VA) at two stations (PM7 and NM3; see map in Fig. 1). (DIN  = NO_3_ + 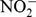 +NH_4_).

Parameters	PM7	NM3
Salinity	28.64	22.40
DIN (µM)	3.42	32.0
PO_4_ (µM)	0.24	0.92
Si(OH)_4_ (µM)	13.7	42.7
DOC (mg L^−1^)	1.12	3.66
BA (cells L^−1^)	2.45×10^9^	4.28×10^8^
VA (particles L^−1^)	2.57×10^10^	9.40×10^9^

**Figure 3:**
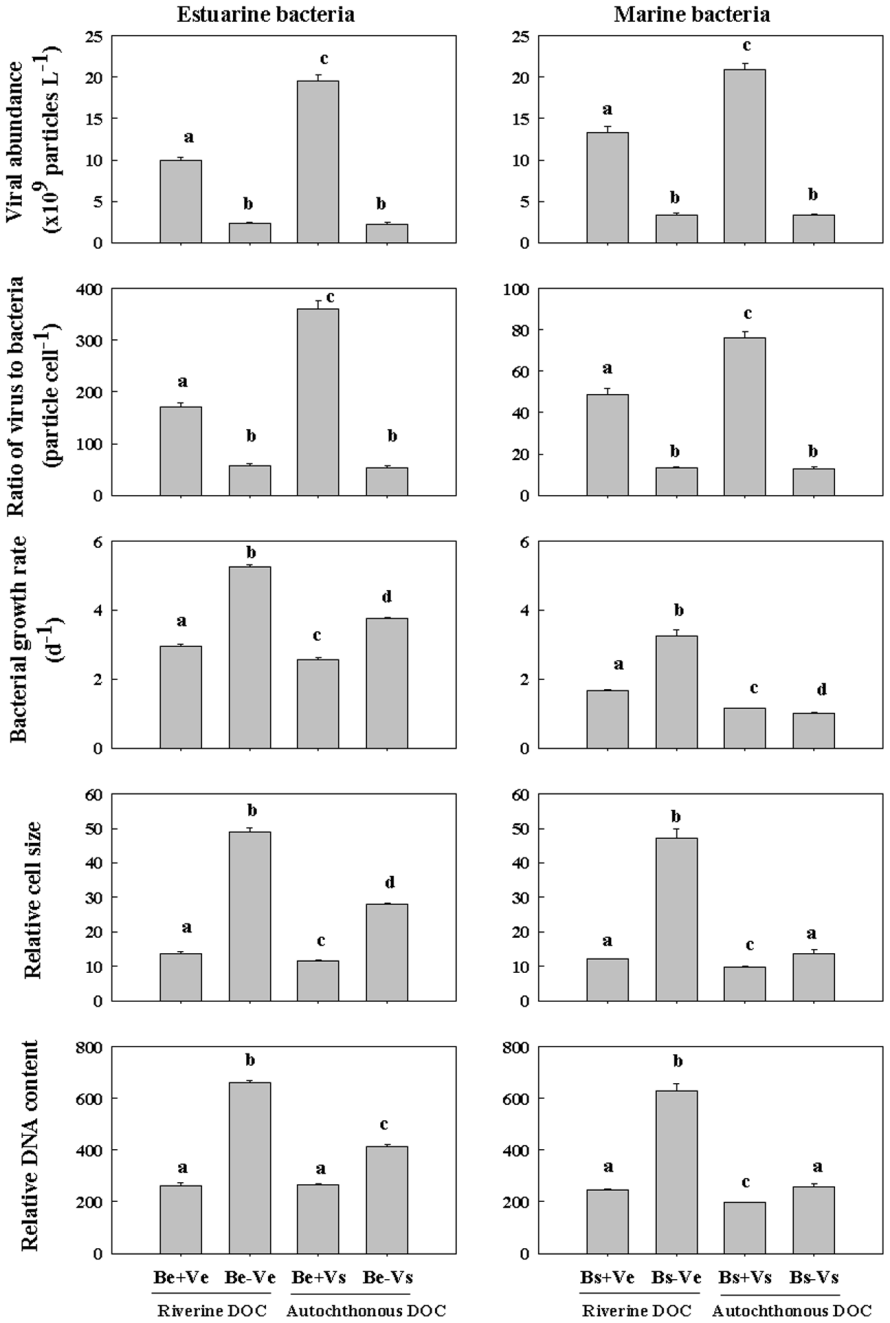
Viral abundance (VA) and virus to bacteria abundance ratio at the beginning of the incubation. Bacterial growth rate, relative cell size, and relative DNA content at the end of the incubation among eight treatments: 1) estuarine bacteria + virus-rich estuarine water (Be+Ve); 2) estuarine bacteria + virus-free estuarine water (Be-Ve); 3) estuarine bacteria + virus-rich seawater (Be+Vs); 4) estuarine bacteria + virus-free seawater (Be-Vs); 5) seawater bacteria + virus-rich estuarine water (Bs+Ve); 6) seawater bacteria + virus-free estuarine water (Bs-Ve); 7) seawater bacteria + virus-rich seawater (Bs+Vs); 8) seawater bacteria + virus-free seawater (Bs-Vs). Vertical bars indicate ± 1SE and n = 3. Different letters (a, b, c, d) denote that the treatment was significantly different (*p*<0.05) and the same letters denote that the treatment was not significantly different (*p*>0.05).

**Figure 4:**
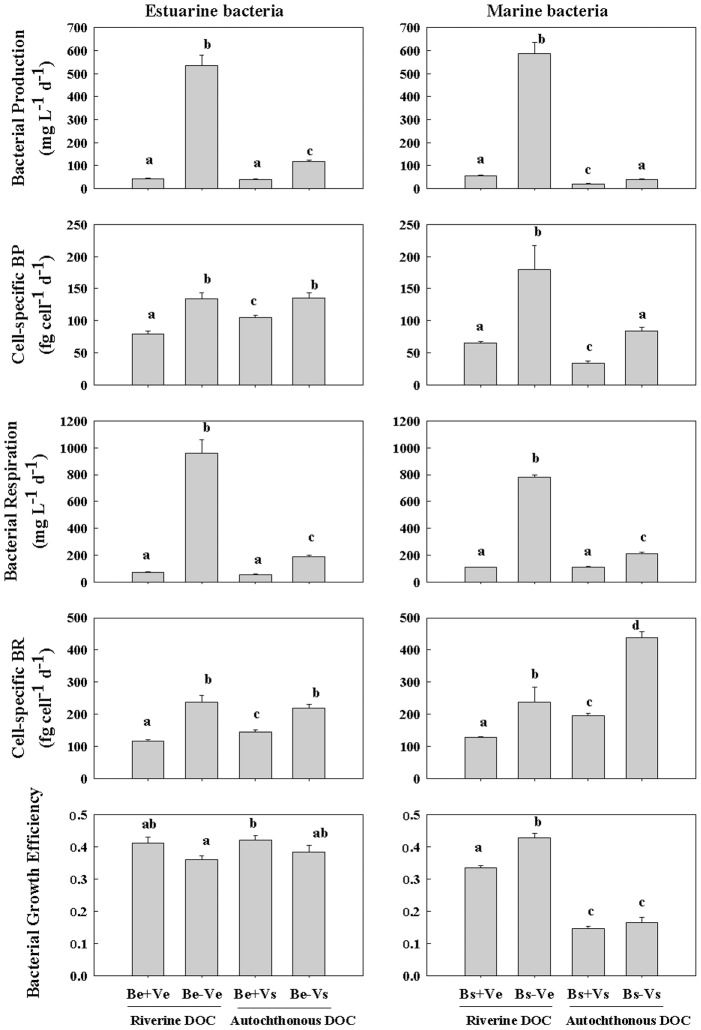
Bacterial production (BP), cell-specific bacterial production, bacterial respiration (BR), cell-specific bacterial respiration and bacterial growth efficiency at the end of the incubation among eight treatments. Error bars indicate ± 1SE and n = 3. Different letters (a, b, c, d) denote that the treatment was significantly different (*p*<0.05) and the same letters denote that the treatment was not significantly different (*p*>0.05). See Fig. 3 for treatment abbreviations.

For the bioassay experiments with estuarine bacteria, viral abundance was the highest (1.95×10^10^ particles L^−1^) in the Be+Vs treatment, moderate (1.00×10^10^ particles L^−1^) in the Be+Ve treatment and the lowest (∼2.20×10^9^ particles L^−1^) in the Be-Ve and Be-Vs treatments (Fig. 3). The abundance ratio of viruses to bacteria followed the pattern of viral abundance. Bacterial growth rates differed significantly among the four treatments, with low growth rates (2.9 and 2.6 d^−1^) in the virus-rich treatment (i.e. Be+Ve and Be+Vs) and high (3.7 and 5.3 d^−1^) in the virus-free treatment (i.e. Be-Ve and Be-Vs). The relative cell size and DNA content of bacteria followed the pattern of bacterial growth rate (Fig. 3). Bacterial production (535 and 118 µg L^−1^ d^−1^) and cell-specific bacterial production (134 and 136 fg cell^−1^ d^−1^) in the virus-free conditions (i.e. Be-Ve and Be-Vs) was significantly higher than the counterpart (42.7 and 40.6 µg L^−1^ d^−1^) and (79.3 and 106 fg cell^−1^ d^−1^) in the virus-rich conditions (i.e. Be+Ve and Be+Vs), respectively. Bacterial production did not differ significantly between the Be+Ve and Be+Vs treatment, while the cell-specific bacterial production in the Be+Vs treatment was significantly higher than that in the Be+Ve treatment. Similarly, bacterial respiration and cell-specific bacterial respiration followed the same patterns as bacterial production and cell-specific bacterial production, respectively (Fig. 4).

For the bioassay experiments with marine bacteria, viral abundance was the highest (2.10×10^10^ particles L^−1^) in the Bs+Vs treatment, moderate (1.33×10^10^ particles L^−1^) in the Bs+Ve treatment and the lowest (∼3.30×10^9^ particles L^−1^) in the Bs-Ve and Bs-Vs treatments (Fig. 3). Bacterial growth rates differed significantly among four treatments, being low (1.1 and 1.0 d^−1^) in the autochthonous DOC treatment (i.e. Bs+Vs and Bs-Vs) and high (1.7 and 3.2 d^−1^) in the riverine DOC treatment (i.e. Bs+Ve and Bs-Ve). The relative cell size and DNA content of bacteria showed the same pattern (Fig. 3). Bacterial production (586 and 39.1 µg L^−1^ d^−1^) and cell-specific bacterial production (178 and 84.4 fg cell^−1^ d^−1^) in the virus-free conditions (i.e. Bs-Ve and Be-Vs) was significantly higher than the counterpart (56.0 and 19.5 µg L^−1^ d^−1^) and (65.4 and 34.5 fg cell^−1^ d^−1^) in the virus-rich conditions (i.e. Bs+Ve and Bs+Vs), respectively. Bacterial production and cell-specific bacterial production in the Bs+Ve treatment were significantly higher than those in the Bs+Vs treatment. Bacterial respiration (783 and 209 µg L^−1^ d^−1^) in the virus-free conditions (i.e. Bs-Ve and Bs-Vs) was significantly higher than that (110 and 112 µg L^−1^ d^−1^) in the virus-rich conditions (i.e. Bs+Ve and Bs+Vs), while bacterial respiration did not differ significantly between the Bs+Ve and Bs+Vs treatment. The cell-specific bacterial respiration (239 and 438 fg cell^−1^ d^−1^) in the virus-free conditions (i.e. Bs-Ve and Bs-Vs) was significantly higher than that (129 and 197 fg cell^−1^ d^−1^) in the virus-rich conditions (i.e. Bs+Ve and Bs+Vs) (Fig. 4).

For the bioassay experiments with marine bacteria, bacterial production and cell-specific bacterial production on the riverine DOC were significantly higher than those on the autochthonous coastal DOC (Stn PM7), irrespective of the presence and absence of viruses. Bacterial respiration on the riverine DOC was significantly higher than that on the autochthonous DOC in the virus-free conditions, but not in the virus-rich conditions. In contrast, the cell-specific bacterial respiration (129 and 239 fg cell^−1^ d^−1^) on the riverine DOC was significantly lower than that (197 and 438 fg cell^−1^ d^−1^) on autochthonous DOC, irrespective of the presence or absence of viruses. Bacterial growth efficiency (0.33 and 0.43) on the riverine DOC was significantly higher than that (0.15 and 0.17) on autochthonous DOC, irrespective of the presence or absence of viruses (Fig.4).

## Discussion

### Response of bacterial metabolic activity to riverine DOC input

The effects of DOC on bacterial metabolic activity are often assessed in the presence of viruses, which might bias the bacterial response to DOC supply alone [37] and underestimate bioavailability of DOC. In this study, a comparison of the bacterial response to riverine DOC between the presence or absence of viruses provided insight into the bioavailability of riverine DOC and the role of viruses in regulating carbon cycling in the Pearl River estuary.

**Figure 5:**
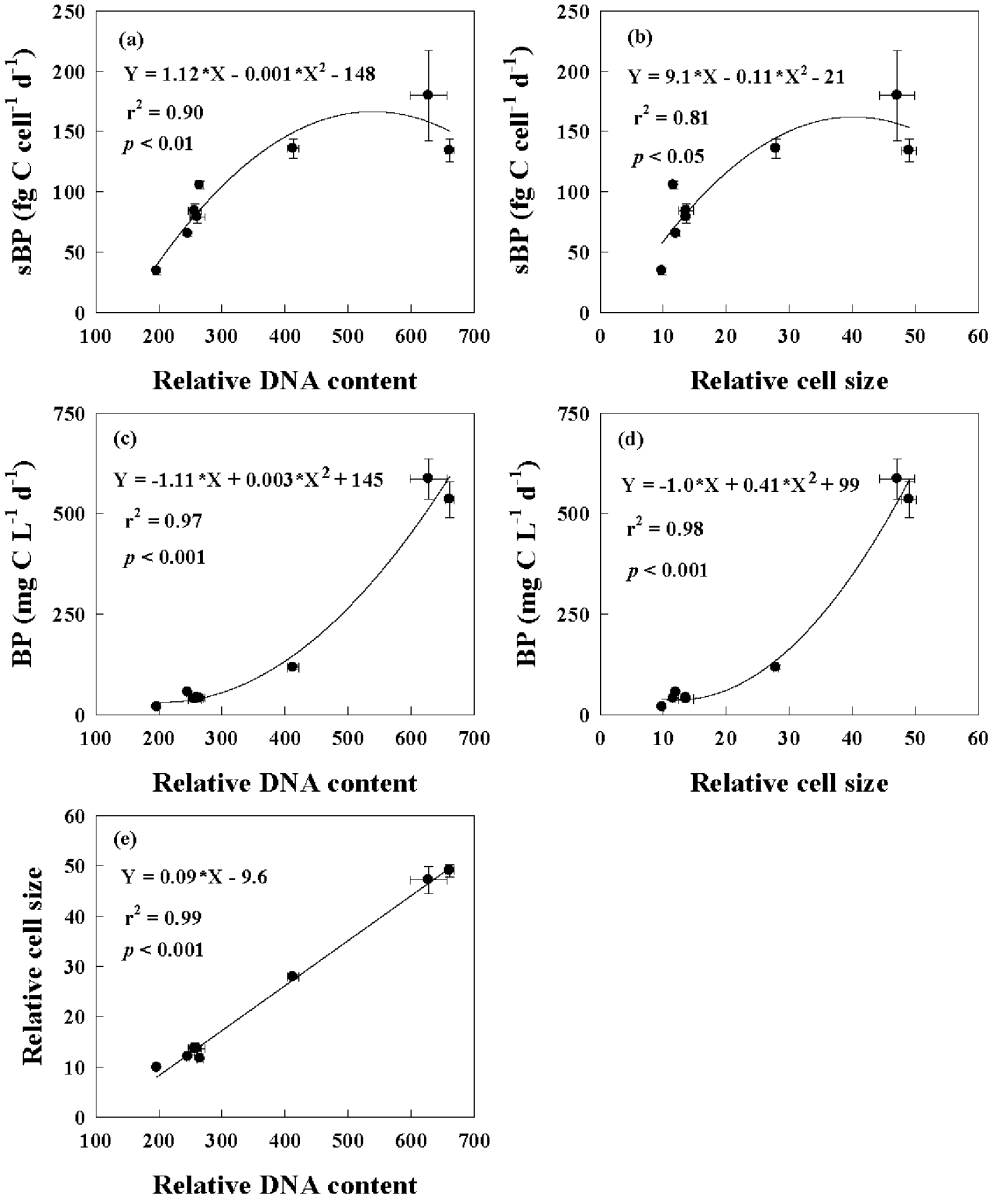
The relationship at the end of the incubation between: (a) cell-specific bacterial production (sBP) vs relative DNA content, (b) cell-specific bacterial production (sBP) vs relative cell size, (c) bacterial production (BP) vs relative DNA content, (d) bacterial production (BP) vs relative cell size, and (e) relative cell size vs relative DNA content for eight treatments.

Our results showed that bacterial metabolic activity responded significantly to the riverine DOC input irrespective of the presence or absence of viruses. Generally, bacterial production, cell-specific BP, growth rate, relative cell size and DNA content were significantly higher in the riverine DOC treatments (e.g. Bs+Ve and Bs-Ve) than the counterpart in the autothchonous coastal DOC treatments (e.g. Bs+Vs and Bs-Vs), respectively. These results suggested that riverine DOC was highly bioavailable and efficiently utilized, which favored the growth of large and metabolically active marine bacteria, especially under virus-free conditions. However, in the presence of viruses, the increased DOC utilization was mitigated considerably by viral lyses, since the large and metabolically active bacteria may have been preferentially killed by viruses [38] and less competitive bacterial species that were resistant to viral infection survived [39], [40]. In addition, the viral shunt is partly responsible for low bacterial production in the presence of viruses [41]. It is estimated that up to 50% of bacterial mortality is due to viral lyses in marine environments [22], [25]. A significant correlation between bacterial production and the relative DNA content or cell size (Fig. 5) further confirmed that viruses played a major role in regulating bacterial production. The stimulation of bacterial production by riverine DOC in the presence of viruses implied that the addition of bioavailable DOC might, to some extent, weaken viral control of bacterial production by improving the capability of the metabolically active bacteria to resist viral lysis. This finding was consistent with previous reports [42], [43].

In response to riverine DOC input, the bulk bacterial respiration was enhanced significantly in the absence of viruses, but not in the presence of viruses, which was contradictory with previous reports that viruses enhanced the role of bacteria as oxidizers of organic matter and producers of CO_2_ by stimulating bacterial respiration [26]. Viral infection has been reported to enhance BR likely due to the decomposition of the viral lysate with an energetic cost [44]. Interestingly, the cell-specific bacterial respiration in the riverine DOC treatments (i.e. Bs+Ve and Bs-Ve) was significantly lower than that in the autochthonous coastal DOC treatments (Bs+Vs and Bs-Vs) irrespective of the presence or absence of viruses, which was the opposite pattern for the cell-specific bacterial production. The cell-specific bacterial respiration has been found to be sensitive to changes in substrates, which increases sharply with the deceasing quality and quantity of the substrates [20], [24]. The high cell-specific bacterial production and low cell-specific bacterial respiration in the riverine DOC treatment further corroborated that riverine DOC was highly labile, resulting in higher bacterial growth efficiency. An early study showed that sewage effluents with high concentrations of carbohydrates and amino acids, which are among the most labile fraction of the bulk organic matter [45], [46], were the major sources (32–54%) of riverine DOC in the Pearl River estuary [8]. Our observations are in agreement with the finding from our previous sewage-seawater cross-transplant experiment that sewage effluents enhanced bacterial production, but not bacterial respiration, resulting in high bacterial growth efficiency [21]. The bioavailable riverine DOC input influenced the bulk bacterial respiration in two contrasting ways; it enhanced the bulk BR by stimulating bacterial growth and reduced the cell-specific BR due to its high lability. Riverine DOC input altered the partitioning between production and respiration within the natural bacterial community, with a larger proportion of carbon used for biomass synthesis and less for maintenance. The high supply of riverine DOC exposed bacteria to labile DOC in the Pearl River estuary with repaid discharge and a short residence time. As a result, there was little stimulation of the bulk BR by riverine DOC. This might partly explain the lower CO_2_ emission in some estuaries that receive a large amount of riverine DOC with a high percentage of sewage-derived DOC. The relatively low bacterial production in the presence of viruses was more likely due to the repression of the metabolically active bacteria, rather than the viral shunt, since the viral shunt would increase bacterial respiration [26], [47].

Viral infection dramatically decreased bacterial carbon demand. Bacterial carbon demand ( = BP+BR) in the presence of viruses only accounted for 7–12% of that in the absence of viruses. The bioavailability of riverine DOC in the presence of viruses was greatly underestimated, since bacterial production was primarily regulated by viruses, rather than DOC [24]. In conventional microbial decomposition experiments, the biodegradable DOC is estimated as the difference between the DOC at the beginning and end of the incubation that could last for several days or up to one month. In this case, the estimated bioavailability of DOC is likely biased because a fraction of the bacterial biomass is returned to the DOC pool as semi-labile or recalcitrant DOC via the viral shunt [48], which might be substantial during a long-term incubation.

### Response of bacterial metabolic activity to exogenous viruses

Shifts in the viral community composition are observed along a salinity gradient in estuaries [49], as well as viral infection rates [50]. To date, little attention has been paid to the composition and metabolic response of the natural bacterial assemblage to varying sources of viruses in estuaries [27]. In our study, bacterial mortality induced by exogenous viruses was assessed through a cross-transplant experiment of estuarine water and seawater. The relative cell size, DNA content, and cell-specific bacterial production in the Be+Vs treatment were significantly lower than that in the Be-Vs treatment, suggesting that marine viruses were able to repress the metabolically active estuarine bacteria. However, the cell-specific bacterial production in the Be+Ve treatment was significantly lower than that in the Be+Vs treatment, despite the high DOC availability in the Be+Ve treatment, implying that marine viruses were able to infect estuarine bacteria, but not as efficiently as estuarine viruses. This was inconsistent with a previous report that marine viruses may not be able to infect freshwater bacteria [27]. In contrast, a 2.7-fold difference in the cell-specific bacterial production for marine bacteria between in virus-free (Bs-Ve) and virus-rich (Bs+Ve) estuarine water was similar to the difference (2.4-fold) between in virus-free (Bs-Vs) and virus-rich (Bs+Vs) seawater. It revealed that estuarine viruses were able to efficiently infect marine bacteria. The same finding has been reported by *Bonilla-Findji et al.* [27]. The rapid changes in the viral community induced by freshwater input into estuaries might alter the existing relationship between bacteria and viruses, leading to changes in bacterial metabolic activity and community composition along a salinity gradient in estuarine environments, in addition to the salinity effect [51], [52]. As the capability of utilizing the various DOC component varies with the type of microorganism [53], the effect of changes in the bacterial community composition and bacterial metabolic activity induced by exogenous viruses on the carbon cycle in estuaries should not be ignored. Further studies are needed to confirm the interaction of bacteria and exogenous viruses.

### Evaluation of the Experimental Approach

Recent studies suggest that salinity may be an important factor regulating virus-host interactions [52], [54]. In this study, estuarine water in the middle section of the Pearl River estuary served as the riverine DOC source instead of freshwater in the upper section of the estuary, in order to reduce the effect of low salinity on the bacterial metabolic activity in our experiments. Our earlier study has shown that large bacteria typically captured on a 1 µm polycarbonate membrane filter were negligible when the bacterial inoculum was prepared [21]. The empirical conversion factor of 3.0 kg C mol leucine^−1^ was adopted to calculate bacterial production and we cannot rule out the possibility that BP for bacteria grown on autochthonous DOC was overestimated, since DOC at coastal station PM7 was at the relatively low level and less labile. The DNA content is a useful proxy for bacterial activity [55]. The significant correlation between cell-specific bacterial production and relative DNA content (Fig. 5) indicated that the estimation of BP based on the commonly used empirical conversion factor was valid.

## Conclusions

Riverine DOC input increased bacterial production significantly, but not bacterial respiration because of its high lability. The bioavailable riverine DOC input influenced the bulk bacterial respiration in two contrasting ways; it enhanced the bulk BR by stimulating bacterial growth and reduced the cell-specific BR due to its high lability. As a result, there was little stimulation of the bulk BR by riverine DOC. The lability of riverine DOC might be partly responsible for lower CO_2_ degassing fluxes in some DOC-rich estuaries. Viruses reduced bacterial carbon demand dramatically by repressing the growth of metabolically active bacteria, which eventually restricted microbial decomposition of riverine DOC. Bacterial carbon demand in the presence of viruses only accounted for 7–12% of that in the absence of viruses. Hence, a significant fraction of riverine DOC was transported offshore to the shelf and slope ocean.
